# Aortopathy in Bicuspid Aortic Valve: Pathophysiology, Risk Stratification and Surgical Decision-Making—A Narrative Review

**DOI:** 10.3390/jcm15093542

**Published:** 2026-05-06

**Authors:** Sebastian Krych, Julia Gniewek, Michał Jurkiewicz, Paweł Kowalczyk, Dariusz Waniczek, Tomasz Hrapkowicz

**Affiliations:** 1 Department of Cardiac, Vascular and Endovascular Surgery and Transplantology, School of Medical Sciences in Zabrze, Medical University of Silesia, Marii Skłodowskiej-Curie 9, 41-800 Zabrze, Poland; thrapkowicz@sum.edu.pl; 2 Student’s Scientific Society, Department of Cardiac, Vascular and Endovascular Surgery and Transplantology, School of Medical Sciences in Zabrze, Medical University of Silesia, Marii Skłodowskiej-Curie 9, 41-800 Zabrze, Poland; julia.m.gniewek@gmail.com; 3 Student’s Scientific Society, III Department of Cardiology, School of Medical Sciences in Zabrze, Medical University of Silesia, Marii Skłodowskiej-Curie 9, 41-800 Zabrze, Poland; jurkiewicz.m@yahoo.com; 4 Department of Animal Nutrition, The Kielanowski Institute of Animal Physiology and Nutrition, Polish Academy of Sciences, Instytucka 3, 05-100 Jabłonna, Poland; 5 Department of Oncological Surgery, Faculty of Medical Sciences in Zabrze, Medical University of Silesia, 40-055 Katowice, Poland; dwaniczek@sum.edu.pl

**Keywords:** aneurysm, ascending aorta, cardiac surgery, connective tissue disease, genetic disorders, biological and socio-demographic risk factors

## Abstract

Bicuspid aortic valve (BAV) is one of the most common congenital heart defects, introducing significant hemodynamic disturbances to the circulatory system. This narrative review analyzed articles published between 2012 and 2025 and indexed in PubMed. The aim was to synthesize key information on the etiopathogenesis of BAV, its potential complications and associated risks, as well as available pharmacological and surgical treatment strategies, with emphasis on indications and contraindications for specific surgical techniques. Analyses demonstrate that isolated BAV with typical valvular aortopathy is associated with a more favorable prognosis compared to valvular aortopathy syndromes. Valve phenotype shows important sex-related relationships in its presentation and progression. From a hemodynamic perspective, BAV alters blood flow angles, which may contribute to weakening of the aortic wall and secondary valve changes. BAV is also frequently associated with genetic disorders such as Marfan syndrome. The heterogeneity of aortopathies linked to BAV creates significant challenges for echocardiographers, cardiologists and cardiac surgeons, particularly in determining the optimal timing and strategy for surgical intervention.

## 1. Introduction

Bicuspid aortic valve (BAV) is the most common congenital aortic valve structural defect, characterized by the presence of two semilunar cusps. It affects 0.5–2% of live births with a male-to-female ratio of 3:1. This defect is associated with a high risk of stenosis and aortopathy involving the aortic root and ascending aorta. Untreated severe aortic valve stenosis is a life-threatening condition. The treatment of choice is cardiac surgery, which involves replacing the diseased valve with a mechanical or biological prosthesis [1].

Bicuspid aortic valve is associated not only with progressive valvular dysfunction and aneurysm formation, but also with a substantially increased lifetime risk of acute aortic syndromes, particularly ascending aortic dissection (Stanford type A). This risk is related both to congenital abnormalities of the aortic media and to chronic abnormal flow patterns generating asymmetric wall shear stress. Early recognition of aortic enlargement and timely surgical referral are therefore essential components of management [2].

**Figure 1:**
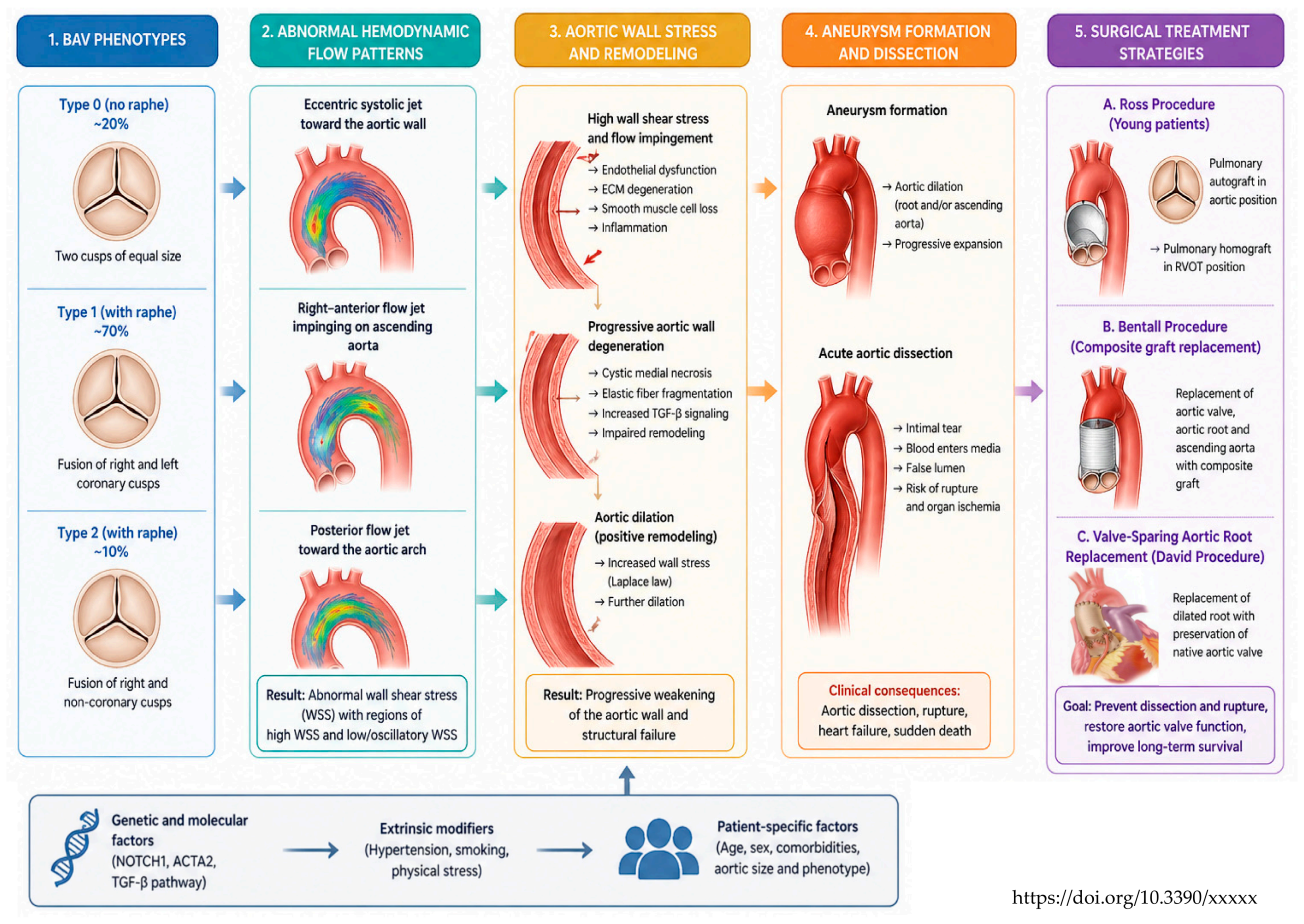
Pathophysiological relationships in bicuspid aortic valve (BAV) disease and corresponding surgical treatment strategies.

For patients at high surgical risk, transcatheter aortic valve implantation (TAVI) is an alternative. In cases where TAVI is not feasible or must be postponed for various reasons, aortic valve valvuloplasty, also known as Balloon Aortic Valvuloplasty (BAV), may be performed. This temporary procedure involves percutaneous insertion and inflation of a balloon to dilate the valve annulus and leaflets. However, balloon valvuloplasty does not permanently restore valve function, with satisfactory effects typically lasting 3–6 months, followed by recurrence of stenosis and symptoms. The procedure is intended to relieve symptoms, improve circulatory function, and provide time for further diagnostics and preparation for definitive treatment, such as valve replacement or TAVI [1,2,3,4,5,6]. BAV causes significant hemodynamic disturbances in aortic blood flow. These abnormal flow patterns are associated with pathological changes at the cellular level, including disorders of the elastic membrane, which impair its adaptation to high blood pressure during left ventricular contraction [1,2,3,4,5,6]. The aim of this narrative review is to summarize the current state of knowledge related to the phenotyping of BAV, the genetic factors predisposing to its development, associated hemodynamic abnormalities, diagnostic methods, and surgical management techniques (Figure 1).

## 2. Methods

**Scheme 1:**
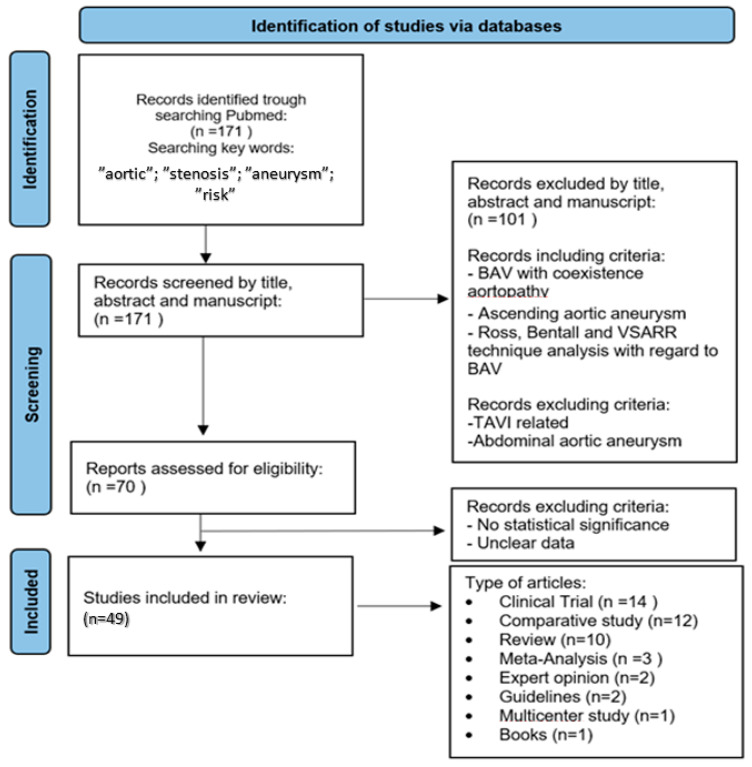
Details of the methodology are outlined in PRISMA 3.

Articles were identified via the PubMed database using the following keywords: aortic, stenosis, aneurysm, and risk. We reviewed articles published from January 2012 to March 2026. The following article types have been included: Clinical Trial, Meta-Analysis, Randomized Controlled Trial, Review, and Systematic Review. Of 171 initially retrieved articles, 70 were selected, and 49 were ultimately included in this review. All studies published before 2012, works related to TAVI, isolated aortic valve surgery, and those focusing on abdominal aortic aneurysms were excluded. Exceptions were made for the following high-value publications: Siu and Silversides (Bicuspid aortic valve disease, 2010) [6] and Svensson et al. (2011) [7]. Inclusion criteria were BAV with coexistence of aortopathy, ascending aortic aneurysm, Ross, Bentall and VSARR technique analysis with regard to BAV. The details of the methodology are outlined in PRISMA (flow Scheme 1). The analysis was not registered on PROSPERO.

## 3. Results

### 3.1. BAV Classification

Depending on the clinical presentation, BAV can be classified into three distinct groups.

The first group, termed valve aortopathy syndrome, is associated with the worst prognosis. Its occurrence may be linked to genetic factors and other coexisting congenital heart defects. Patients in this group are at high risk of severe aortic stenosis, rapid progression of valve dysfunction, and aortopathy. It mainly affects children and young adults, often necessitating early surgical intervention due to the rapid progression of the defect.

The second group, typical valvular aortopathy, is characterized by slower progression and milder symptoms compared to group 1. This form is usually diagnosed in adults, with surgical intervention typically required at a later time.

The third group includes patients with undiagnosed and uncomplicated congenital valve defects. These are long-term conditions, with moderate or non-progressive valvular aortopathy. The absence of clinical signs frequently leads to incidental discovery during diagnostic tests or autopsy. Once diagnosed, further evaluation is necessary to determine the appropriate management and potential treatment [1,2,3,4,5,6].

### 3.2. Types and Phenotypes of Bicuspid Aortic Valve

Type 1 BAV (fusion) is the most common, with a prevalence of 90–95%. It is characterized by three aortic sinuses, two cusps of symmetrical or asymmetrical shape and size, and a connective tissue strand often present between the cusps.

Type 2 (two sinuses) is less common, with a prevalence of 5–7%. It has two aortic sinuses, two cusps of symmetrical shape and size, without a connective tissue band. Two commissures can be observed.

Type 3 BAV (partial fusion) has an undefined prevalence. It consists of three aortic sinuses, three symmetrical cusps and three visible joints, one of which may be fused. A small connective tissue band may be present between the cusps [2,6,8].

All phenotypes of BAV fusion are summarized in Table 1.

### 3.3. Gender-Specific Phenotypic Differences

In men, the aortic annulus, sinus, sinotubular junction, and ascending aorta are generally slightly larger, on average by 3 mm, than in women. Fusion of the right and left cusps is more common in men (approximately 12.5% higher), whereas fusion of the right and non-coronary cusps is more frequent in women (approximately 12.5% higher). Men are also more likely to develop aortic stenosis, while women more commonly present with aortic regurgitation [9,10].

### 3.4. Average Aortic Root Measurements: BAV (Bicuspid Aortic Valve) vs. TAV (Tricuspid Aortic Valve)

BAV is associated with distinct aortic root dimensions compared to TAV, which should be considered when qualifying patients for surgical procedures. CT measurements were obtained in patients scheduled for aortic valve replacement, including 40 patients with BAV and 72 with TAV. On average, aortic root dimensions were smaller in patients with BAV than in those with TAV. These differences underscore the importance of accounting for anatomical variability in BAV when planning optimal surgical treatment. Average aortic root measurements for BAV and TAV are presented in Table 2.

### 3.5. Hemodynamics of the Bicuspid Valve in Relation to the Normal Tricuspid Valve

The bicuspid valve has a significantly higher mean pressure gradient. Late systolic and late diastolic cardiac volume and left ventricle ejection fraction (LVEF) are not significantly affected. However, higher left ventricle mass index (LVMI) values have been observed in BAV patients (mean 30 g/m^2^) [12]. Phenotypic differences and associated hemodynamic disturbances are closely linked to the risk of aortopathy. Blood flow through type 2 BAV is relatively central, tending to curve towards the left coronary sinus. The effective orifice area (EOA) for this type averages 1.84 cm^2^, and the ejected blood volume does not directly impact the aortic wall, which is associated with a lower risk of aortic dilation. In contrast, types 1 and 3 BAV are less favorable hemodynamically, as the ejected blood fraction is directed away from the central axis, increasing stress on the aortic wall and the risk of aortopathy.

Type 1 BAV has an approximate EOA of 1.77 cm^2^ [13]. In the left-right cusp fusion (L/R) phenotype, blood flow is directed obliquely to the anterior-lateral wall of the ascending aorta. In contrast, the right-noncoronary cusp fusion (R/N) phenotype directs flow toward the anterior-posterior wall of this segment of the aorta [3]. No significant differences have been observed between these two phenotypes regarding indexed aortic diameter or the incidence of severe aortic pathologies [3]. Chandran et al. [14] have reported that the R/N phenotype predisposes to dilation of the ascending aorta and a potentially higher risk of stenosis, despite lower radial contraction velocity and acceleration along the anterior wall of the ascending aorta [14]. Additionally, a correlation exists between the angle of the blood stream and the indexed aortic diameter: an LV-aorta angle > 50° is associated with a higher frequency of indexed diameter ≥ 22 mm/m^2^, whereas angles < 50° are less frequently associated with an indexed diameter ≥ 22 mm/m^2^ [3].

Type 3 BAV has the smallest EOA of the three types, averaging 1.60 cm^2^, and is associated with significantly impaired flow hemodynamics. The strongly eccentric orientation of the ejection blood fraction causes it to continuously impinge upon and flow along the outer wall of the ascending aorta. This atypical stress also places the highest mechanical load on the valve cusps, resulting in a greater tendency for cusp damage and accelerated degeneration [13].

### 3.6. Genetic Factors Associated with Increased Risk of Bicuspid Valve Development

The etiology of the bicuspid aortic valve has a genetic background. Loci on chromosomes 1p21 and 2q22 have been associated with BAV development, particularly through deletions in the GATA 4, GATA 6, NOTCH1 and NKX2. 5 HD genes. Dysfunction of the GATA gene family leads to general abnormalities in the development of cardiac cells originating from the neural crest. The heterozygous E386X mutation in GATA 6 is characteristic of autosomal dominant BAV inheritance [4]. NOTCH1 mutations, disrupting neural crest cell migration during valvulogenesis, are strongly associated with BAV and increase the risk of valve calcification and aortic aneurysm formation [15,16].

#### 3.6.1. Syndromes Associated with BAV

BAV occurs in several defined genetic syndromes, including Marfan syndrome (*FBN1* mutation), Loeys-Dietz syndrome (*TGFBR2* mutation), Bosley Salih–Alorainy syndrome (*HOXA 1* mutation), and Andersen syndrome (*KCNJ2* mutation). Marfan and Loeys-Dietz syndromes additionally predispose patients to the development of aneurysms, Bosley Salih–Alorainy is linked to other congenital heart defects (like ventricular septal defect, and tetralogy of Fallot), while Andersen syndrome is associated with aortic coarctation and pulmonary valve stenosis [15].

#### 3.6.2. SOD Gene Mutation

Changes in the expression of the *SOD* gene encoding the enzyme superoxide dismutase are strongly associated with the type and phenotype presented by the valve, as well as the presence or absence of aneurysm lesions.

The *SOD1* gene is overexpressed in type 1 left-right BAV fusion, with increased expression in the left ascending aorta associated with aortic obstruction. In contrast, *SOD2* expression does not show significant changes, while *SOD3* expression is reduced in type 1 left-right BAV fusion. Although the impact of reduced *SOD3* expression on the development of aortic stenosis remains uncertain, it cannot be excluded.

In BAV with ascending aorta aneurysm, *SOD1* overexpression is observed in type 1 left-right and right-noncoronary BAV fusion, localized respectively to the left or noncoronary region of the aneurysm. A decrease in *SOD2* expression occurs in type 1 left-right BAV fusion, and reduced *SOD2* has been associated with valve insufficiency. Insufficient *SOD3* expression is characteristic of type 1 right-noncoronary BAV fusion and type 2 BAVs. Since similar patterns have been observed in other aortic pathologies, these findings are not unique to BAV [17]. Analysis of changes in SOD gene expression may help explain the embryogenesis of specific BAV phenotypes. This will allow for estimating the probability of developing a bicuspid valve defect and planning a diagnostic and treatment path.

### 3.7. Aortopathy in BAV

BAV is one of the major accompanying conditions associated with disease of the ascending aorta. Lesions are most frequently localized in the ascending aorta or the aortic root [18,19,20,21]

#### 3.7.1. Definition of Aortopathy

Aortopathy encompasses disorders of the aorta characterized by progressive weakening of the vessel wall. These conditions can be determined by genetic or mechanical causes, and both factors influence the course of BAV. Gradual widening of the aorta promotes the formation of aneurysms. As the vessel wall becomes thinner, the risk of its dissection or rupture increases. Such events often cause massive hemorrhage and are frequently fatal. In the general population, the prevalence of aortic aneurysms is estimated at 5.9 cases/100,000 people [18].

#### 3.7.2. Most Common Locations of Aortic Aneurysms

Significant widening of the aorta in a given section increases the risk of aortic aneurysm. The most common site of this widening is the ascending aorta, affecting 49% of children and 27% of adults with BAV [19,20]. No correlation has been observed between the frequency of this widening and gender [20,21]. Dilation of the aortic root is less frequent, affecting 22% of pediatric patients [20] and 6.1% of adults [21]. In adults, prevalence is higher in men (7.6% vs. 1.2%) [21]. A relationship exists between the type of BAV fusion and the site of aortic dilation. Fusion of the right and left cusp is associated with dilatation extending from the aortic root to the proximal arch, while right–noncoronary cusp fusion is more frequently linked to ascending aortic dilatation, which may progress to involve the arch and root [8].

#### 3.7.3. Developmental Factors

The aorta shows loss of a concentric layer of smooth muscle cells (SMCs) on a non-inflammatory substrate. The mechanism of degeneration helps differentiate the etiology of aortopathy in BAV and Marfan syndrome. In the former case, immature SMCs produce an insufficient number of Fbn-1 fibers, while in the latter, Fbn-1 mutations promote SMC apoptosis [4].

#### 3.7.4. Genetic Factors

Genetic factors account for approximately 20% of all aortic aneurysms [18]. The genotype of the KLK1 rs5516 locus also plays a role. Homozygosity for the D allele has been associated with a significantly higher incidence of aortic aneurysms. The frequency of genotypes with G alleles is higher in patients with aortic aneurysms compared to the control group, and homozygosity for the G allele correlates with the coexistence of hypertension and aortic aneurysm [19].

#### 3.7.5. Physiological Factors

Physiological factors contributing to aortopathy include matrix metalloproteinases (MMPs), whose increased activity is characteristic of the BAV condition. This leads to degradation of extracellular matrix components such as fibronectin, collagen, elastin and proteoglycans. Eventually, the process weakens the aortic wall, predisposing to aneurysm formation. Although tissue inhibitors of metalloproteinases (TIMPs) act as a compensatory mechanism, deregulation of the MMP/TIMP balance results in loss of this protective effect. Patients with BAV and dilation of the ascending aorta show increased MMP-2 activity [4].

#### 3.7.6. Risk of Rupture of Aortic Aneurysm During BAV

The risk of rupture or dissection of an aortic aneurysm, and the associated risk of sudden death, must be assessed in the context of the patient’s overall clinical profile. Patients diagnosed with BAV and coexisting genetic disorders such as Marfan or Loeys-Dietz syndromes are at particularly high risk, requiring surgical intervention at smaller aortic diameters of 4.0 cm and 4.5 cm, respectively. In isolated BAV, a less restrictive approach is possible, with intervention generally considered at ≥5.5 cm, or 5.0 cm in patients with a family history of dissection or rapid aneurysm progression [22]. Dilation involving the aortic root (often accompanied by the ascending aorta) usually has a poorer prognosis, as it is associated with faster diameter progression and a greater risk compared with lesions confined to the root [23]. The rate of aneurysm growth is also slower in women compared to men [24,25]. Another critical factor is the relationship between diameter and wall thickness; as the aorta widens, its walls become thinner, increasing the risk of dissection or rupture [26]. Traditional risk factors for aneurysms, such as hypertension and dyslipidemia, are less relevant in BAV compared to TAV patients, as BAV aortopathy has a strong genetic component [27]. Consequently, risk assessment must be individualized, taking into account not only aortic diameter but also additional features such as involvement of the aortic root or ascending aorta. While current threshold values for surgical intervention provide useful guidance, they remain imperfect predictors of rupture risk.

### 3.8. Diagnosis

BAV diagnosis is based on transthoracic echocardiography (TTE), transesophageal echocardiography (TEE), and cross-sectional imaging with computed tomography (CT) or cardiac magnetic resonance (CMR). These imaging methods also allow for precise measurements of the aortic root and ascending aorta [6,9].

### 3.9. Pharmacological Treatment

Pharmacological treatment is an alternative to surgery, aiming to slow the progressive enlargement of the aorta and stabilize the patient’s hemodynamic status. The most commonly used drugs are beta-blockers, which lower blood pressure and reduce both the rate of aneurysm growth and the risk of dissection [22]. Their effectiveness depends on the type and phenotype of BAV. More asymmetric blood flow through the valve increases wall shear forces in the ascending aorta, and in such cases, beta-blockers may not achieve the expected reduction in risk [28]. Plaque stabilization and anticoagulant therapy are recommended to lower the risk of myocardial infarction, while statins in patients with hypercholesterolemia reduce the incidence of cardiovascular and cerebrovascular events. Losartan and angiotensin-converting enzyme inhibitors (ACEi) have shown particular benefits in Marfan syndrome in terms of reducing aortic risk. In addition to pharmacotherapy, patients should be advised to quit smoking, manage diabetes, and maintain a healthy body weight [22].

### 3.10. Surgical Treatment

Qualification for the procedure requires collaboration among specialists in cardiac surgery, pediatric cardiac surgery, and anesthesiology. Evaluation includes analysis of the overall clinical picture, risk assessment using the STS-PROM and EuroSCORE II scales. The patient’s nutritional status, cognitive function, comorbidities (e.g., severe pulmonary disease), and anatomical features, including porcelain aorta or other anatomical malformations, are also taken into account [29].

#### 3.10.1. Technique

Cardiac surgery is selected according to the patient’s clinical presentation and age. Each surgical approach differs in terms of procedure duration, type of access, and potential risks and complications. The most commonly used surgical techniques are described in the following sections.

#### 3.10.2. Ross Procedure

First described by Donald Ross in 1967, this procedure is mainly used in pediatric patients but may also be applied to adults. The technique involves replacing the diseased bicuspid aortic valve with a transplant of the patient’s own pulmonary valve. The resulting pulmonary valve deficit is reconstructed using a homograft, such as Contegra or Freestyle conduit. The prepared coronary ostia are reattached to the graft or allograft as required [30].

#### 3.10.3. Bentall Procedure

Bentall surgery for patients with BAV involves two main steps. First, the coronary arteries are detached from the aortic bulb at the level of the coronary ostia, leaving an 8–12 mm margin of the arterial wall. Subsequently, the bicuspid valve is excised and replaced with either a biological prosthesis (BBP/Bio-Bentall-Procedure) or a mechanical prosthesis (MBP/Mechanical-Bentall-Procedure). The pathologically dilated segment of the aorta is resected and a vascular graft is sutured in its place. Preassembled grafts combining a mechanical valve with a vascular conduit may also be used [31,32]. An alternative to full median sternotomy is a partial inverted type “J” sternotomy, referred to as the “Mini-Bentall” procedure. It provides access to the ascending aorta and improves visualization of the aortic arch when hemi-arch replacement is required [33]. This minimally invasive approach is associated with faster healing of the sternum and a quicker return to the patient’s preoperative activity level.

#### 3.10.4. David Procedure (VSARR)

The David procedure, or valve-sparing aortic root replacement (VSARR), is another surgical technique used in patients with BAV aortopathy. Several modifications of the David procedure exist, all sharing the replacement of the ascending aorta with a vascular graft, preservation of the patient’s native valve, and reimplantation of coronary ostia.

The original David I technique developed by Tirone David involves reimplanting the patient’s native valve into a vascular graft, securing it within the ventriculo-aortic junction, attaching the valve flaps to the newly formed commissures, and finally reimplanting the coronary ostia.

The Yacoub technique (David II) preserves the native valve and commissures while removing the walls forming the Valsalva sinuses. Truncated vascular graft tongues replace the removed sinuses. Valve closure is corrected by adjusting the leaflets through precise stitching and tensioning, followed by reimplantation of the coronary ostia.

The David III technique is similar to Yacoub (David II) but additionally incorporates external annuloplasty of the aortic ring to prevent subsequent dilatation.

David IV uses larger vascular grafts (diameter > 2–4 mm). In this modification, the upper margin of the reconstructed aortic root is additionally sutured to reduce its diameter.

The David V technique employs 4–8 mm larger vascular grafts than standard and includes stitching to narrow both the upper and lower margins of the reconstructed root [34].

The Stanford modification of David V uses two vascular grafts. The larger graft (6–8 mm wider than standard dimensions) is sutured at all three commissures to create pseudo-sinuses. The distal section is narrowed, while the proximal segment is adjusted according to the valve morphology. In cases of BAV or a significantly widened aortic ring, the bottom of the graft is also narrowed to correct the ring’s diameter. If the ring size is already correct, only a minor adjustment is needed for a proper fit. The graft is then secured at the ventriculo-aortic junction, and the patient’s native valve cusps are sutured to the newly formed commissures. The second, smaller graft is attached to the distal ascending aorta, matching the diameter of the narrowed distal margin of the larger graft. Finally, the two grafts are joined, and the coronary ostia are reimplanted [35].

VSARR had some technique modifications. Miller et al. describe the VSARR reimplantation technique. The main part of the surgery was extended to a virtual basal ring resection of the aortic root. Coronary ostia must be prepared and cut off from the sinuses of valsalva. In the last step, the native BAV war reimplanted with a dacron graft in the commissural position. Calcification or fibrous tissue on the reimplanted valve was removed. Both of the free margin of the bicuspid valve cusp was trimmed through the plication technique. It is possible to repair the valve and regain coaptation using a pericardial patch [36].

#### 3.10.5. Isolated AVR/AV Plastic Surgery

Isolated replacement or plastic surgery of the aortic valve in the course of obstruction or BAV stenosis is performed after assessment of the condition of the ascending aorta, both at the stage of qualification for the procedure and intraoperatively. Initially, the possibility of repairing the defective aortic valve is assessed, followed by the decision to replace it with a biological or mechanical prosthesis, depending on the overall clinical picture [37]. Due to the fact that this review focuses on BAV and associated aortopathies, which almost always require intervention on the widened section of the aorta, isolated AVRs and AV plastic surgery will not be discussed further.

### 3.11. Indications and Contraindications for Surgical Treatment

#### 3.11.1. Ross Procedure

Originally, this technique was reserved mainly for pediatric patients, but it is now increasingly used in adults, typically under 50 years of age. In selected cases, it may also be considered in patients aged 50–65, provided that the benefits outweigh the potential risks [7,37,38]. Patients over 50 years of age being considered for the Ross procedure should meet additional criteria [7,38]. All criteria are presented in Table 3.

It should be emphasized that a widening of the ascending aorta ≥ 40 mm with a normal aortic annulus is not an absolute contraindication for the Ross procedure. In such cases, the surgical technique is modified by replacing the dilated aortic segment with a Dacron graft, which significantly reduces the subsequent risk of pulmonary homograft valve failure [38].

#### 3.11.2. Bentall Procedure

The Bentall procedure is a surgical technique for replacing the aortic valve together with the aortic arch, mainly in adults. The average age of qualified patients ranges from 50 to 60 years, with a predominance of men. Patients with Marfan syndrome represent an important subset of those qualified [39].

Most contraindications for the Bentall procedure are relative. Since access is usually obtained via median sternotomy, the surgical approach can be adapted based on intraoperative conditions. Contraindications mainly apply to modifications such as the Mini-Bentall procedure, where limited surgical access may pose technical challenges, and the implantation of mechanical or biological valves, in line with current ESC guidelines [7,34,35,36,37,38,39,40,41]. All indications and contraindications are summarized in Table 4.

#### 3.11.3. David Procedure (VSARR)

David’s procedure is primarily performed in adults, with the average age of patients ranging from 50 to 55 years and a predominance of males. This method is applicable for BAV-associated aortopathies as well as for aortopathies related to genetic connective tissue disorders, such as Marfan syndrome [34]. Key indications and contraindications for the David procedure are presented in Table 5.

The Yacoub technique (David II) requires specific consideration. While it provides a better hemodynamic profile and more natural leaflet function compared to the David I technique, it is also associated with a higher risk of aortic ring dilation, particularly in patients with connective tissue disorders such as Marfan syndrome [34]. David IV, V and V, and Stanford-modified David V are currently the most promising techniques. They allow the creation of Valsalva pseudosinuses, which mimic the blood flow and physiology of a healthy aortic root. Theoretically, the pseudosinuses reduce the closure rate of the cusps and stress on the cusps during the diastolic phase, potentially minimizing mechanical damage and extending the valve’s lifespan. However, no studies have yet confirmed these assumptions, and extensive prospective observations will be required to validate them [35].

For the VSARR reimplantation technique modification has one critical criterion. Nativ BAV must be repairable. Coexisting aortic valve stenosis with calcification was a contraindication for this surgery type [36].

A summary table directly comparing the primary surgical techniques is presented in Table 6.

### 3.12. Surgical Treatment Risk and Mortality

#### 3.12.1. Ross Procedure

Current data on intra-operative mortality during the Ross procedure remain inconsistent, with studies reporting a risk ranging from 1% to 3.2%. This procedure is still relatively uncommon, and the risk of death is closely correlated with the number of surgeries performed at the facility and the experience of the cardiac surgery team, indicating a significant learning curve. The annual rate of surgical reinterventions per patient is estimated at 0.5–1.5%, meaning that 85–95% of patients do not require reoperation within 10 years [7]. Kari et al. analyzed outcomes in 258 patients undergoing Ross surgery and 1444 undergoing AVR hospitalized at the same center. In a matched cohort of 208 pairs, major risk factors, including intraoperative mortality and reoperation rates, were comparable between groups. However, Ross patients had a significantly lower risk of hemorrhagic strokes and major bleeding, which was directly attributed to the absence of long-term antiplatelet therapy. In addition, these patients were much less likely to die from heart-valve-related causes [43].

#### 3.12.2. Bentall Procedure

The average EuroSCORE risk for the Bentall procedure ranges from 2% to 6%. However, it should be noted that BAV patients are on average 7 years younger than those with TAV, and more than 13 years younger when admitted for acute dissection, with significantly fewer comorbidities overall. There are no significant differences in the 30-day survival between patients with BAV (both isolated and Marfan syndrome) and patients with TAV [31,39,40]. The difference lies in the causes of postoperative death of patients. For patients with BAV, the most frequent causes are multi-organ failures and serious brain injuries (reported in the source as “brain injuries” without further specification), while in TAV patients, the predominant cause is low cardiac output syndrome [40]. The incidence of postoperative complications does not differ significantly between BAV and TAV patients. The most common complications for both groups are respiratory failure, bleeding requiring a second surgery, heart rhythm disorders requiring a pacemaker, and gastrointestinal complications [40].

#### 3.12.3. David Procedure (VSARR)

A large meta-analysis by Mazine et al. [44] compared valve-sparing aortic root replacement in TAV versus BAV patients and demonstrated a similar risk of in-hospital mortality for both groups (0.00% vs. 1.93%; *p* = 0.11). Moreover, the rate of hospital reoperation was also comparable (5.64% vs. 5.99%; *p* = 0.98). However, the five-year mortality rate was significantly lower for BAV patients than in those with TAV after the David procedure [1.63% vs. 8.15%; *p* = 0.02). On the other hand, reintervention rate due to aortic insufficiency was significantly higher for BAV patients at 1-, 3-, 5- and 10-year follow-ups. However, a detailed analysis excluding the first 3 years of follow-up showed no significant differences in reintervention rates between BAV and TAV (0.55% vs. 0.59%; *p* = 0.64) after 3–5 years and (1.80% vs. 1.17%; *p* = 0.06) after 5 years [44].

The average risk associated with the David procedure is similar to that of the Bentall procedure. Patients selected for the David procedure are on average 6 years younger and demonstrate a more favorable cardiac profile, with higher NYHA functional class (≥II), better LVEF (58% ± 0.08 vs. Bentall 0.53 ± 0.12), and lower aortic obstruction (mean ˂ 2). No significant differences in 30-day mortality or reoperation due to bleeding are observed postoperatively. Patients undergoing David surgery show greater improvement in LVEF (average +4%), and lower peak gradients at the aortic valve (average −4 mmHg). What is more, the David surgery is associated with a significantly lower risk of stroke and TIA. Over a six-year period, freedom from reintervention on the aortic valve and ascending aorta is similar for both the David and Bentall procedures [32].

Zuo et al. in a Danish multicentre nationwide study about VSARR demonstrate that valve morphology has no effect on the risk of reoperation with relation to VSARR or Bental technique and BAV 7.4% or TAV 5.2%, morphology (*p* = 0.20) [45].

Retention of the native valve in patients with BAV morphology, including those with concomitant Marfan syndrome, markedly prolongs the period of normal valve function and increases mean survival over a 14-year scale compared to biological valve implantation. While Marfan syndrome is associated with a poorer overall prognosis, the period of normal valve function without intervention is still longer than for patients with an isolated BAV or TAV [42].

Overall survival rate in the VSARR reimplantation technique was 97.6% after 5 years (95% CI, 96.2–99.1%), and 93.8% (95% CI, 89.9–96.2%) after 10 years. In addition, overall freedom from reoperation at 1, 5, and 10 years after surgery was as follows: 99.1% (95% CI, 98.6–99.8%); 95.4% (95% CI, 92.7–97.1%), and 89.2% (95% CI, 83.4–93.1%) [36].

## 4. Discussion 

This narrative review, based on a structured literature search, summarizes the current state of knowledge regarding aortopathy associated with bicuspid aortic valve (BAV), with particular emphasis on its complex etiopathogenesis, phenotypic variability, and diagnostic and therapeutic implications. BAV, as the most common congenital valvular heart defect, is a significant predisposing factor for the development of aortic pathologies, including dilation of the aortic root and ascending aorta, the formation of aneurysms, and aortic dissection. Aortopathy associated with BAV is not a homogeneous condition; rather, it results from the coexistence of several overlapping mechanisms [1,2,3,4,5,6]. Its clinical course depends on the interaction between the aortic valve phenotype, hemodynamic disturbances, genetic factors, and individual clinical factors. Abnormal blood flow patterns, increased shear stress on the aortic wall, extracellular matrix remodeling, and developmental structural abnormalities of the vessel wall are of crucial importance [2]. In clinical practice, this means that the evaluation of a patient with BAV should take into account not only anatomical parameters but also the broader biological, hemodynamic, and clinical context. Modification of the clinical picture and slowing of disease progression are only partially achievable through blood pressure reduction with ACE inhibitors and stabilization of atherosclerotic plaques with statins. Unfortunately, drug therapy merely delays the need for surgical intervention. The use of imaging techniques such as TTE, CT, and CMR should not be limited only to confirming the diagnosis but should constitute the basis for a dynamic assessment of the risk of disease progression [8]. This can aid in selecting the optimal therapeutic strategy, taking into account the patient’s clinical condition, the presented valvular phenotype, the degree of aortic dilatation, and the limitations associated with specific surgical techniques.

This overview of the etiopathogenesis, risks, and treatment of aortopathy associated with a bicuspid aortic valve is not exhaustive. Ongoing studies, including genetic research, continue to provide new insights. The multifactorial and relatively unpredictable course of the disease requires constant monitoring and implementation of therapeutic procedures in the early stages of developing aortopathy. It is also necessary to take into consideration the latest findings in molecular biology and the predisposition to specific BAV phenotypes, as well as the mechanisms underlying hemodynamic disturbances [10,13,15]. Modern pharmacotherapy can effectively slow the progression of aortic dilatation for a considerable period, but surgical intervention almost invariably becomes necessary at some point.

Procedures previously reserved for pediatric patients, such as the Ross procedure, are increasingly being applied in adults. Nevertheless, the Bentall and David procedure remains the gold standard for managing aortopathy in patients with a bicuspid aortic valve (BAV). These techniques are continuously improved to reduce risk factors, improve postoperative predictability, and ultimately increase patient survival rates [29].

Despite new findings in the field of etiopathogenesis, predisposing factors for the defect, and the influence of the phenotype on the profile of hemodynamic abnormalities, BAV raises many unanswered questions for clinicians. Further clarification and research are needed to understand the correlations between the BAV phenotype, potential biomarkers, and surgical qualification criteria.

However, it should be emphasized that despite advances in operational techniques, many aspects still require detailed investigation, including the safety of surgical access, postoperative mortality, reintervention risk, and the incidence of neurological complications in both BAV and TAV patients. To improve procedural safety for patients, it is essential to improve risk models and influence the presented phenotypic and therapeutic profiles.

In the context of bicuspid aortic valve (BAV)-associated aortopathy, increasing attention has been directed toward the complex interplay between oxidative stress, inflammatory response, and disturbances in hemostasis, which collectively determine disease progression and the risk of complications. As highlighted by Ravn et al., oxidative stress plays a pivotal role in initiating DNA damage and activating cellular response pathways, ultimately leading to chronic inflammation and degeneration of the aortic wall [46]. These mechanisms may account for the observed phenotypic heterogeneity of aortopathy in BAV patients, where the rate of aortic dilation does not consistently correlate with hemodynamic factors alone but is also influenced by individual molecular susceptibility.

An important extension of this perspective is the concept of spatial heterogeneity in thrombus formation, as proposed in the study on the Total Thrombus-Formation Analysis System (T-TAS). The authors suggest that local variations in flow conditions and vascular wall properties may influence coagulation activation and platelet function, which could be relevant for assessing the risk of complications such as aortic dissection or rupture [47]. The incorporation of tools evaluating dynamic thrombogenic properties may, in the future, complement conventional risk stratification models that are currently based predominantly on aortic dimensions.

Furthermore, systemic factors such as obesity and its associated prothrombotic state may modulate the course of aortopathy. Gniewek et al. emphasize that obesity is linked to enhanced coagulation activation, endothelial dysfunction, and chronic low-grade inflammation, all of which may accelerate degenerative processes within the aortic wall [48,49]. In the context of BAV, this underscores the need for a more holistic patient assessment, integrating not only anatomical parameters but also metabolic and inflammatory profiles.

From a surgical decision-making standpoint, these observations highlight the limitations of current criteria based solely on aortic diameter. The integration of biomarkers of oxidative stress, hemostatic parameters, and metabolic factors may enable more precise identification of high-risk patients who could benefit from earlier surgical intervention. Thus, the development of multifactorial approaches appears crucial for optimizing the management of patients with BAV and associated aortopathy.

The timing of prophylactic surgical intervention in ascending aortic disease should not be based exclusively on absolute aortic diameter thresholds. Although current guideline cut-offs remain essential references, clinical decision-making is increasingly supported by a broader individualized risk assessment. Parameters such as rapid aortic enlargement, bicuspid aortic valve phenotype, predominant root involvement, positive family history of dissection, underlying connective tissue disorders, and evidence of inflammatory or metabolic activity may indicate increased vulnerability despite dimensions below conventional thresholds. In addition, patient age, comorbidities, and estimated operative risk should be considered when balancing procedural benefit against surgical burden. Therefore, optimal timing of intervention is best determined within a multidisciplinary heart team framework integrating imaging, clinical, and patient-specific factors.

In the future, the integration of molecular, imaging, and clinical data may enable more precise patient risk stratification, better prediction of disease progression, and selection of interventions. Advances in imaging techniques, predictive tools, and surgical treatment methods are expected to improve treatment safety and the prognosis for patients with BAV. Addressing these challenges will be the task of the next generation of cardiac surgeons and scientists.

## 5. Limitations

This study has several limitations based on the narrative analysis character. Potentially risk of bias during the selection and data analysis process. To minimize risk, the selection of publications for analysis was carried out by an impartial three-member committee, with each member voting to include or exclude a publication from the analysis based on their academic expertise and the established search criteria. The analysis was not registered on PROSPERO.

Potentially risk of subjectivity due to strict search criteria focused on BAV with coexisting aortopathy.

Scope restriction was minimized over a wide range of search years from 2012 to 2026.

## 6. Future Directions and Research

Artificial Intelligence may significantly enhance future management of BAV-associated aortopathy through automated multimodality imaging analysis, personalized risk prediction, and integration of genomic and clinical datasets. AI-assisted tools may improve the timing of surgery and help identify patients at high risk despite subthreshold aortic diameters.

## 7. Conclusions

Bicuspid aortic valve-associated aortopathy is a multifactorial disorder resulting from the interaction of congenital wall abnormalities, genetic predisposition, and abnormal hemodynamic stress. Risk assessment should extend beyond aortic diameter alone and incorporate phenotype, growth rate, root involvement, and patient-specific factors. Surgical management remains the cornerstone of treatment, with Ross, Bentall, and valve-sparing root replacement representing key options in selected patients.

The decision regarding operative timing in ascending aortic disease should follow a personalized, multiparametric approach rather than rely solely on aortic diameter. Integrating anatomical features, disease progression, genetic and clinical risk markers, and operative risk may improve patient selection and support safer, more effective management. Multidisciplinary heart team evaluation remains central to this strategy.

Future integration of advanced imaging, biomarkers, and Artificial Intelligence may enable more personalized and safer treatment strategies.

## Figures and Tables

**Table 1:** Types of bicuspid aortic valve (BAV) and corresponding phenotypes [2,6,8].

Types of BAV	Type 1	Type 2	Type 3
Phenotypes	Fusion of right and left cusp	Lateral-Lateral	Partial fusion of one joint
	Fusion of right cusp with non-coronary cusp	Anterior-Posterior	
	Fusion of left cusp with non-coronary cusp		
	Unspecified cusps fusion		

**Table 2:** CT aortic root measurements in patients qualified for cardiac surgery aortic valve replacement [11].

Aortic Root Measurements [11]		
Average Measurements	BAV	TAV
Minimal root dimension [mm]	41.3 ± 7.4	45 ± 8.8
Root area [cm^2^]	15.8 ± 5.3	20 ± 12.4
Sinotubular junction [mm]	40.3 ± 6.5	44.1 ± 9.9
Ascending aorta [mm]	51.9 ± 8.6	50 ± 11.3

(BAV—bicuspid aortic valve; TAV—tricuspid aortic valve).

**Table 3:** Ross procedure indications, contraindications, and additional criteria for patients over 50 [7,38].

**Ross procedure in patient under 50 years**	
**Indication**	**Contraindication**
Planned pregnancy	Aortic annulus diameter ≥ 27 mm
High level of physical activity	Risk of mismatch if AoV/PV diameter difference ˃ 2 mm
Contraindications to antiplatelet therapy	Co-existing autoimmune diseases significantly affect pulmonary homograft durability
Favorable anatomical conditions	Dilation of the aortic annulus with concomitant aortic insufficiency due to hereditary aortopathy
Infective endocarditis of the aortic valve	
**Additional Ross procedure criteria for patients over 50 (in addition to standard indications for patients under 50)**	
Life expectancy of at least 15 years	
Suitable anatomical configuration	
Absence of severe cardiovascular or concomitant diseases (e.g., chronic kidney disease on dialysis, radiation-induced valvular disease)	
Active lifestyle	

**Table 4:** Indications and contraindications for Bentall and Mini-Bentall procedures [34,39,40,41].

Bentall procedure criteria	
**Indication**	**Contraindication**
Ectasia of the aortic ring	Contraindications are relative. If possible, a minimally invasive Mini-Bentall with partial sternotomy is preferred. Disqualification from Mini-Bentall is also a contraindication for the standard Bentall procedure
Acute or chronic dissection of the aorta	
Significant widening of the aortic root and ascending aorta	
Failure/inability to apply the David procedure	
Mini-Bentall procedure contraindication criteria	
Urgent or rescue surgery	
Prior cardiac surgery	
Severe chest deformities	
Infective endocarditis	
Need for additional procedures (MVR, TVR, CABG, etc.)	

**Table 5:** David’s procedure indications and contraindications [32,34,42].

**David’s procedure**
**Indication**
Age < 65 years
No or minimal comorbidities (including cardiovascular)
High LVEF (promotes faster degeneration of biological prostheses)
Procedure to prevent implantation of a biological prosthesis (due to patient age) or a mechanical prosthesis (due to inability to use antiplatelet therapy)
**Contraindication**
Age > 65 years
Damage to the aortic valve leaflets
Calcification of aortic valve leaflets
Anatomical malformations
Prior myocardial ischemia/need to reduce extracorporeal circulation time

**Table 6:** Comparing the three major surgical approaches discussed in the manuscript: Ross procedurę, Bentall procedure, Valve-Sparing Aortic Root Replacement (David procedure/VSARR).

Surgical Technique	Typical Patient Profile (“Gold Standard”)	Preferred Age Group	Main Indications	Major Contraindications/Limitations	Main Advantages	Principal Risk Factors/Concerns	Anticoagulation Required	Reintervention Profile
**Ross** **procedure**	Young, active patients with aortic valve disease, preserved ventricular function, desire to avoid prosthetic valve and anticoagulation; women planning pregnancy	Children, adolescents, adults < 50 years (selected 50–65 years)	Severe aortic stenosis/regurgitation with suitable anatomy; young patients requiring a durable physiological valve substitute	Aortic annulus dilation, significant hereditary aortopathy, mismatch between aortic and pulmonary valve diameters, severe comorbidities, and poor pulmonary valve anatomy	Excellent hemodynamics, living autograft with growth potential, no lifelong anticoagulation, favorable quality of life, lower thromboembolic risk	Technically demanding, two-valve procedure, autograft/root dilation, homograft degeneration, center experience dependent	**No**	Moderate long-term reintervention risk (autograft or homograft related) [7,38,43]
**Bentall procedure**	Adults with a combined aortic root/ascending aorta aneurysm plus valve disease; patients requiring definitive root replacement	Usually 50–70 years (younger in connective tissue disease/dissection)	Root aneurysm, ascending aortic aneurysm with valve pathology, acute/chronic dissection, failed prior valve/root surgery	Frailty/high operative risk (relative), severe comorbidity, limited suitability for minimally invasive access in complex cases	Established gold standard, durable root replacement, excellent control of aneurysm/dissection pathology, reproducible results	Prosthetic valve-related complications, bleeding, stroke, infection, lifelong anticoagulation if a mechanical valve is used	**Mechanical: Yes**/**Bioprosthetic: No**	Low–moderate depending on prosthesis type and patient age [31,32,33,39,40,41]
**Valve-Sparing Aortic Root Replacement (David/VSARR)**	Younger or middle-aged patients with root aneurysm and repairable native valve cusps, especially those wishing to avoid prosthesis	Usually <65 years	Root aneurysm with preserved or repairable BAV function, selected hereditary aortopathy, and younger low-comorbidity patients	Calcified cusps, severe cusp degeneration, advanced stenosis, irreparable leaflet pathology, poor surgical anatomy	Preserves native valve, avoids anticoagulation, superior hemodynamics, lower prosthesis-related complications, and good long-term survival in expert centers	Technically complex, risk of late aortic insufficiency, reoperation if repair fails, surgeon experience is crucial	**No**	Moderate; mainly due to recurrent regurgitation or valve failure [32,34,35,36,42,44,45]
**Isolated AVR (mechanical or bioprosthetic)**	Patients with predominant valvular disease and no significant root/ascending aortic pathology	Broad age range depending on the prosthesis choice	Severe stenosis/regurgitation without major aortic dilation	Significant concomitant aortopathy requiring root/ascending repair	Simpler operation than root replacement, standardized outcomes	Residual untreated aortopathy progression, prosthesis-related complications	Depends on prosthesis type	Depends on prosthesis durability [29,37]

## Data Availability

No new data were created or analyzed in this study.

## Abbreviations

The following abbreviations are used in this manuscript:

BAV	Bicuspid aortic valve
TAV	Tricuspid aortic valve
LVEF	Left ventricle ejection fraction
LVMI	Left ventricle mass indexed to body surface area (g/m^2^)
LV	Left Ventricular
EOA	Effective orifice area
VSD	Ventricular septal defect
TOF	Tetralogy of Fallot
TTE	Transthoracic echocardiography
TEE	Transesophageal echocardiography
CT	Computer tomography
CMR	Cardiac magnetic resonance
AA	Aortic aneurysm
SMCs	Smooth muscle cells
MMPs	Matrix metalloproteinases
TIMPs	Tissue metalloproteinase inhibitors
ACEi	Angiotensin converting enzyme inhibitors
STS-PROM	The Society of Thoracic Surgeons (STS) Predicted Risk of Mortality (PROM)
EuroSCORE II	The European System for Cardiac Operative Risk Evaluation
BBP	Bio-Bentall Procedure
MBP	Mechanical Bentall Procedure
VSARR	Valve sparing aortic root replacement
AVR	Aortic valve replacement
AV plastic surgery	Aortic valve plastic surgery
AoV	Aortic valve
PV	Pulmonary valve
ESC	The European Society of Cardiology
MVR	Mitral valve replacement
TVR	Tricuspid valve replacement
CABG	Coronary artery bypass grafting
NYHA	New York Heart Association
TIA	Transient ischemic attack
T-Tas	Total Thrombus-Formation Analysis System
